# Supplementary material for: The Culturable Mycobiota of Sediments and Associated Microplastics: From a Harbor to a Marine Protected Area, a Comparative Study
Source: J Fungi (Basel). 2022 Aug 31;8(9):927. doi: 10.3390/jof8090927 (PMC9501098; doi:10.3390/jof8090927)
Supplement: Supplementary file 1 [file jof-08-00927-s001.zip › Figure S1.pdf]

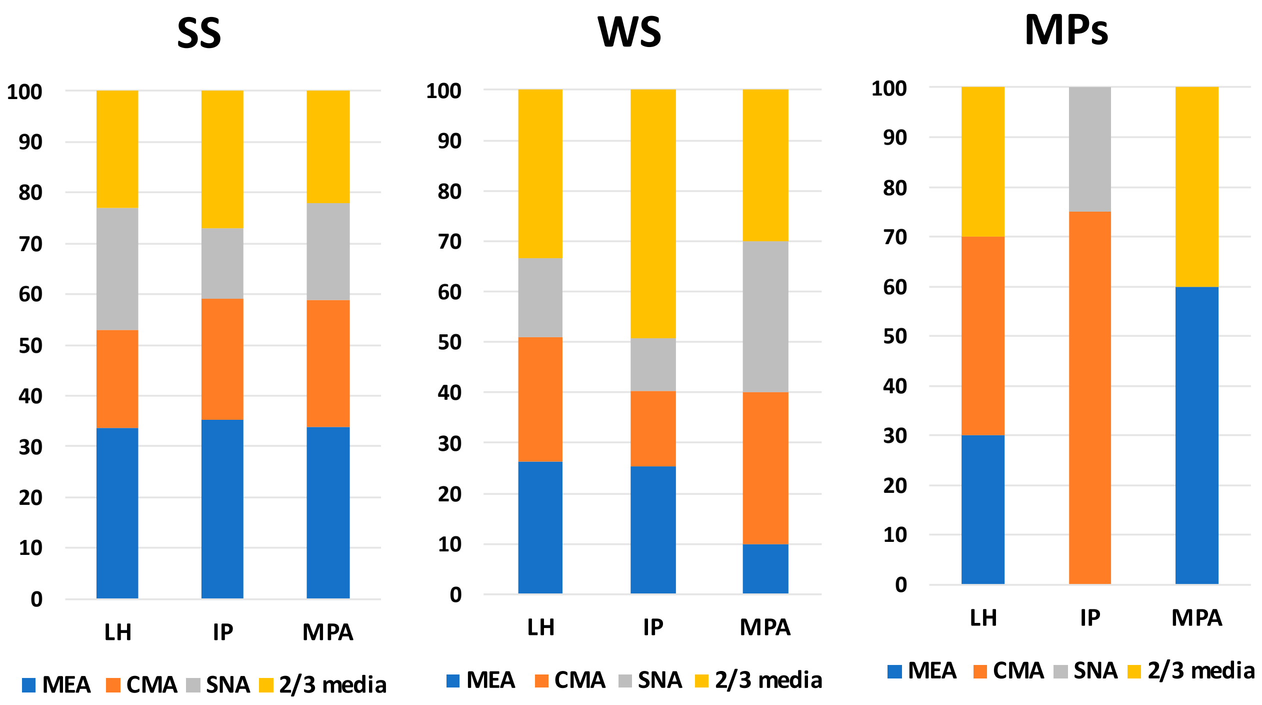

Figure S1 Growth media taxa yield for solid sediments (SS), watery sediments (WS) and sonication of microplastics (MPs) at three different sites: Livorno Harbour (LH), Intermediate point (IP) and Marine Protected Area (MPA), respectively.
