# Supplementary material for: The Culturable Mycobiota of Sediments and Associated Microplastics: From a Harbor to a Marine Protected Area, a Comparative Study
Source: J Fungi (Basel). 2022 Aug 31;8(9):927. doi: 10.3390/jof8090927 (PMC9501098; doi:10.3390/jof8090927)
Supplement: Supplementary file 1 [file jof-08-00927-s001.zip › Table S1.pdf]

Table S1. Average particle size of sediments, components in %.

Sample*	Gravel	Very coarse sand	Coarse sand	Medium sand	Fine sand grain	Very fine sand grain	Pelite
	2000 μm	1000 μm	500 μm	250 μm	125 μm	63 μm	<63 μm
LH	3.52	1.68	1.46	2.86	25.99	33.73	30.76
IP	1.42	1.32	2.07	15.35	58.45	20.79	0.60
MPA	16.52	30.02	52.04	1.34	0.06	0.02	0.00

* Sample: Livorno harbour (LH), intermediate point (IP) and marine protected area (MPA).
