# Supplementary material for: The Culturable Mycobiota of Sediments and Associated Microplastics: From a Harbor to a Marine Protected Area, a Comparative Study
Source: J Fungi (Basel). 2022 Aug 31;8(9):927. doi: 10.3390/jof8090927 (PMC9501098; doi:10.3390/jof8090927)
Supplement: Supplementary file 1 [file jof-08-00927-s001.zip › Table S2.pdf]

Table S2. Trace elements in sediments of Livorno harbour (LH), intermediate point (IP) and marine protected area (MPA), respectively.

Parameter	U.O.M. d.w.*	LH	IP	MPA	LOQ*
Al	%	1.52	0.22	0.10	0.03
As	mg/Kg	6.27	2.86	30.55	1.00
Cd	mg/Kg	0.06	< LOQ	0.03	0.02
Ba	mg/Kg	361.45	4.62	3.93	1.00
Co	mg/Kg	7.52	< LOQ	1.62	1.00
Cr	mg/Kg	61.84	10.33	5.64	1.00
Cu	mg/Kg	48.86	1.88	2.55	1.00
Fe	%	1.94	0.37	0.38	0.03
Mg	%	1.02	0.30	1.21	0.03
Mn	mg/Kg	424.44	168.62	456.82	1.00
Ni	mg/Kg	42.77	8.43	2.11	1.00
P	mg/Kg	562.45	85.30	175.31	5.00
Pb	mg/Kg	15.54	2.74	8.81	1.00
V	mg/Kg	33.23	6.91	22.44	1.00
Zn	mg/Kg	248.50	11.06	12.33	1.00
Hg	mg/Kg	0.10	0.01	0.01	0.005

* U.O.M. = unit of measure, LOQ= limit of quantifications
