# Supplementary material for: The Culturable Mycobiota of Sediments and Associated Microplastics: From a Harbor to a Marine Protected Area, a Comparative Study
Source: J Fungi (Basel). 2022 Aug 31;8(9):927. doi: 10.3390/jof8090927 (PMC9501098; doi:10.3390/jof8090927)
Supplement: Supplementary file 1 [file jof-08-00927-s001.zip › Table S3.pdf]

Table S3. Organopollutants in sediments of Livorno harbour (LH), intermediate point (IP) and marine protected area (MPA), respectively. (LOQ, limit of quantification; U.O.M = unity of measurement)

Parameter	U.O.M. d.w.	LH	IP	MPA
IPA tot	µg/Kg	577.2	10.1	2.9
Acenaphtene	µg/Kg	< LOQ	1.0	0.9
Acenaphthylene	µg/Kg	4.1	< LOQ	< LOQ
Anthracene	µg/Kg	3.9	< LOQ	< LOQ
Benzo[a]anthracene	µg/Kg	40.4	0.5	< LOQ
Benzo[a]pyrene	µg/Kg	51.2	< LOQ	< LOQ
Benzo[b]fluoranthene	µg/Kg	120.4	1.2	< LOQ
Benzo[ghi]perylene	µg/Kg	53.2	0.6	< LOQ
Benzo[k]fluoranthene	µg/Kg	43.4	0.5	< LOQ
Chrysene	µg/Kg	45.8	< LOQ	< LOQ
Dibenz[a,h]anthracene	µg/Kg	34.5	0.8	< LOQ
Fluoranthene	µg/Kg	55.9	0.7	< LOQ
Fluorene	µg/Kg	< LOQ	1.2	0.7
Indeno[1,2,3-c,d]pyrene	µg/Kg	81.2	1.1	< LOQ
Naphthalene	µg/Kg	< LOQ	1.5	< LOQ
Phenanthrene	µg/Kg	4.6	1.1	1.4
Pyrene	µg/Kg	38.4	< LOQ	< LOQ
PCB tot	µg/Kg	19.1	< LOQ	< LOQ
PCB 52	µg/Kg	2.8	< LOQ	< LOQ
PCB 95	µg/Kg	1.9	< LOQ	< LOQ
PCB 99	µg/Kg	1.1	< LOQ	< LOQ
PCB 101	µg/Kg	1.1	< LOQ	< LOQ
PCB 110	µg/Kg	2.8	< LOQ	< LOQ
PCB 123	µg/Kg	1.5	< LOQ	< LOQ
PCB 138	µg/Kg	2.2	< LOQ	< LOQ
PCB 149	µg/Kg	1.9	< LOQ	< LOQ
PCB 153	µg/Kg	< LOQ	< LOQ	< LOQ
PCB 160	µg/Kg	3.9	< LOQ	< LOQ
Hydrocarbons C >10	mg/Kg	116.5	9.9	6.6
Hydrocarbons C< 10	µg/Kg	12.6	17.9	< LOQ

Parameter	U.O.M. d.w.	LH	IP	MPA
IPA tot	µg/Kg	577.2	10.1	2.9
Acenaphtene	µg/Kg	< LOQ	1.0	0.9
Acenaphthylene	µg/Kg	4.1	< LOQ	< LOQ
Anthracene	µg/Kg	3.9	< LOQ	< LOQ
Benzo[a]anthracene	µg/Kg	40.4	0.5	< LOQ
Benzo[a]pyrene	µg/Kg	51.2	< LOQ	< LOQ
Benzo[b]fluoranthene	µg/Kg	120.4	1.2	< LOQ
Benzo[ghi]perylene	µg/Kg	53.2	0.6	< LOQ

Benzo[k]fluoranthene	μg/Kg	43.4	0.5	< LOQ
Chrysene	μg/Kg	45.8	< LOQ	< LOQ
Dibenz[a,h]anthracene	μg/Kg	34.5	0.8	< LOQ
Fluoranthene	μg/Kg	55.9	0.7	< LOQ
Fluorene	μg/Kg	< LOQ	1.2	0.7
Indeno[1,2,3-c,d]pyrene	μg/Kg	81.2	1.1	< LOQ
