# Supplementary material for: The Culturable Mycobiota of Sediments and Associated Microplastics: From a Harbor to a Marine Protected Area, a Comparative Study
Source: J Fungi (Basel). 2022 Aug 31;8(9):927. doi: 10.3390/jof8090927 (PMC9501098; doi:10.3390/jof8090927)
Supplement: Supplementary file 1 [file jof-08-00927-s001.zip › Table S4.pdf]

Table S4. Concentration (items/kg) of microplastics in relation to their shape (A) and colors (B).

A)

	Filament	Fragment	Film	Styrofoam
**LH**	26.3±10.6	2.3±3.2	1.0±1.7	0
**IP**	33.3±4.0	2.0±1.7	0.3±0.6	0.3±0.6
**MPA**	31.0±11.0	0.7±1.2	0.7±1.2	0

B)

	Black	Blue	White Transp.	White Opaque	Red	Green	Other
**LH**	6.3±6.0	7.7±1.5	5.3±5.9	3.7±6.4	0.7±1.2	1.0±1.0	1.3±1.5
**IP**	5.3±5.0	11.3±3.5	11.3±5.0	4.0±6.1	0.7±1.2	3.0±1.0	1.3±0.6
**MPA**	11.0±5.6	11.0±5.3	0.7±1.2	0.0±0.0	3.7±2.5	2.7±1.5	3.7±2.5
