# Supplementary material for: The Culturable Mycobiota of Sediments and Associated Microplastics: From a Harbor to a Marine Protected Area, a Comparative Study
Source: J Fungi (Basel). 2022 Aug 31;8(9):927. doi: 10.3390/jof8090927 (PMC9501098; doi:10.3390/jof8090927)
Supplement: Supplementary file 1 [file jof-08-00927-s001.zip › Table S6.pdf]

Table S6. Fungal taxa from Livorno harbour (LH), intermediate point (IP) and Marine Protected Area (MPA), isolated from solid sediment (SS) and watery sediment (WS). Relative abundance (RA%) are reported. Common taxa of both matrices are in bold.

**Taxa**	**LH**		**IP**		**MPA**	
	**SS**	**WS**	**SS**	**WS**	**SS**	**WS**
Acremonium antarcticum (Spegazzini) D. Hawksworth			0.23			
Acremonium pilosum Giraldo, Guarro, Cano & Gene	0.29		2.04			
Acremonium sclerotigenum (Moreau & R. Moreau ex Valenta) W. Gams	0.15					
Acremonium tubakii W. Gams	0.44		0.23			
***Acrostalagmus luteoalbus* (Link) Zare, W. Gams & Schroers**	10.40	0.73	1.13		0.90	
Albifimbria verrucaria (Alb. & Schwein.) L. Lombard & Crous						0.18
***Alternaria alternata* (Fr.) Keissl**.	0.73			0.26		0.06
Alternaria sp.				0.01		
Amesia nigricolor (L.M. Ames) X. Wei Wang & Samson	1.46					
Annulohypoxylon multiforme (Fr.) Y.M. Ju, J.D. Rogers & H.M. Hsieh	0.29					
Apiospora arundinis (Corda) Pintos & P. Alvarado		0.07				
Apiospora marii (Larrondo & Calvo) Pintos & P. Alvarado		0.07				0.06
Apiospora stipae Crous & R.K. Schumach.			0.11			
Apiotrichum dulcitum (Berkhout) Yurkov & Boekhout				0.26		
Apiotrichum laibachii (Windisch) Yurkov & Boekhout				0.68		
Armillaria mellea (Vahl) P. Kumm.					0.90	
Arthopyrenia salicis A. Massal.			0.11			
Arthrinium arundinis (Corda) Dyko & B. Sutton			0.23		0.90	
Arthrinium kogelbergense Crous				0.26		
Arthrinium marii Larrondo & Calvo			0.23		1.35	
***Arthrinium phaeospermum* (Corda) M.B. Ellis**			0.23	0.09		0.53
Ascochyta sp.						0.06
Ascomycot*a* sp.1	0.12					
Ascomycota sp.2	0.12					
Ascomycota sp.3	0.12					
Ascomycota sp.4	0.12					
Ascomycota sp.5	0.30					
Aspergillus aureolatus Munt.-Cvetk. & Bata	0.15					
Aspergillus europaeus Hubka, A. Nováková, Samson, Houbraken, Frisvad, M. Kolařík				0.09		
Aspergillus flavipes (Bainier & Sartory) Thom & Church	0.15					
Aspergillus fumigatus Fresen.	1.02		0.34			
Aspergillus germanicus Frisvad, Varga & Samson			0.23			
***Aspergillus heyangensis* Z.T. Qi, Z.M. Sun & Yu X. Wang**	0.59	0.13	0.68		0.45	0.29
***Aspergillus insuetus* (Bainier) Thom & Church**	0,29			0.09		
Aspergillus jensenii Jurjević, S.W. Peterson & B.W. Horn			0.11			
Aspergillus niger Tiegh.			0.11		1.80	
Aspergillus pseudodeflectus Samson & Mouch.	0.44					
Aspergillus pseudoglaucus Blochwitz	0.73					
Aspergillus sp.			0.11			
Aspergillus spelaeus A. Nováková, Hubka, M. Kolařík & S.W. Peterson				0.09		

Aspergillus tabacinus Nakaz., Y. Takeda, Simo & A. Watan.	0.73					
Aspergillus templicola Visagie, Hirooka & Samson	0.29					
Aspergillus terreus Thom	3.37		0.11			
Aspergillus tubingensis Mosseray		0.07		0.17		
Aspergillus ustus (Bainier) Thom & Church	0.15				0.45	
Aspergillus versicolor (Vuill.) Tirab.	0.59					
***Aureobasidium pullulans* (de Bary) G. Arnaud**		0.86	0.11	0.85	1.35	
Auxarthron conjugatum (Kuehn) G.F. Orr & Kuehn					0.45	
Auxarthron thaxteri (Kuehn) G.F. Orr & Kuehn	0.15					
Beauveria bassiana (Bals.-Criv.) Vuill.			1.13		0.95	
Beauveria brongniartii (Sacc.) Petch					0.05	
Beauveria felina (DC.) J.W. Carmich.	1.61					
Beauveria varroae S.A. Rehner & R.A. Humber			0.11			
Biatriospora sp. (MUT 4883)					0.90	
Bionectriaceae sp			0.23			
Bjerkandera adusta (Willd.) P. Karst.					0.90	
Brunneomyces brunnescens (W. Gams) Giraldo, Gene & Guarro			1.70		1.67	
Cadophora luteo-olivacea (J.F.H. Beyma) T.C. Harr. & McNew			0.11			
Candida membranifaciens (Lodder & Kreger) Wick. & K.A. Burton					0.45	
***Candida norvegica* (Reiersöl) S.A. Mey. & Yarrow**	0.59	0.53				
Candida pseudolambica M.T. Sm. & Poot	0.59		0.34		0.59	
Candida sp.	0.15					
Cephalotrichum asperulum (J.E. Wright & S. Marchand) Sand.-Den., Guarro & Gené				0.09		
***Cephalotrichum columnare* (H.J. Swart) S.P. Abbott**			0.11	0.26		
Cephalotrichum gorgonifer (Bainier) Sand.-Den., Gené & Guarro	0.15					
Cephalotrichum inflatum Y.L. Jiang & T.Y. Zhang		0.20				
Cephalotrichum stemonitis (Pers.) Nees			0.45			
Chaetomium cochliodes Palliser				0.17		
Chaetomium globosum Kunze			0.57			
Chrysosporium lobatum Scharapov					0.45	
Cistella sp.			0.11			
***Cladosporium aggregatocicatricatum* Bensch, Crous & U. Braun**			0.45		0.45	0.06
***Cladosporium allicinum* (Fr.) Bensch, U. Braun & Crous**	0.44	0.07	1.25		2.25	
Cladosporium asperulatum Bensch, Crous & U. Braun	0.73		0.23		0.45	
***Cladosporium cladosporioides* (Fresen.) G.A. de Vries**	0.88	0.33		0.68	0.90	1.12
***Cladosporium halotolerans* Zalar, de Hoog & Gunde-Cim.**		0.59	0.34	0.68		24.28
Cladosporium inversicolor Bensch, Crous & U. Braun						0.12
***Cladosporium limoniforme* Bensch, Crous & U. Braun**	0.44		0.68	0.60		0.47
***Cladosporium perangustum* Bensch, Crous & U. Braun**	0.15	0.07	0.11	0.17	4.96	
***Cladosporium pseudocladosporioides* Bensch, Crous & U. Braun**			1.93	0.85	4.10	0.71
***Cladosporium psychrotolerans* Zalar, de Hoog & Gunde-Cim.**		0.07	0.11		1.80	0.06
Cladosporium ramotenellum K. Schub., Zalar, Crous & U. Braun		0.33		0.17		8.19
Cladosporium sp.2			1.02			
Cladosporium sp.1			1.02		5.82	
Cladosporium sphaerospermum Penz		0.07				

Cladosporium tenellum K. Schub., Zalar, Crous & U. Braun				0.26		
Cladosporium velox Zalar, de Hoog & Gunde-Cim.				0.34		
Cladosporium westerdijkiae Bensch & Samson						0.12
Cladosporium xylophilum Bensch, Shabunin, Crous & U. Braun		0.13				
***Clonostachys rosea* (Link) Schroers, Samuels, Seifert & W. Gams**			0.79	0.43		0.12
Clonostachys rossmaniae Schroers			0.23			
Clonostachys sp.			0.11			
Coniochaeta sp.			0.11			
Cyberlindnera saturnus (Klöcker) Minter	0.15					
***Debaryomyces hansenii* (Zopf) Lodder & Kreger**		0.20		0.34	0.45	
Debaryomyces marasmus Di Menna			0.23			
Diaporthe columnaris (D.F. Farr & Castl.) Udayanga & Castl.			0.11			
Dichotomopilus funicola (Cooke) X. Wei Wang & Samson	0.44		1.81		0.95	
Discula destructiva Redlin	0.44					
Doratomyces microsporus (Sacc.) F.J. Morton & G. Sm.						0.06
Emericella pluriseminata Stchigel & Guarro	0.29					
Emericellopsis maritima Beliakova			0.11			
Emericellopsis minima Stolk	0.59					
Epicoccum nigrum Link					0.45	
Eupenicillium crustaceum F. Ludw.	0.59		0.68		0.54	
Exophiala equina (Pollacci) de Hoog, Vicente, Najafzadeh, Harrak, Badali & Seyedmousavi			0.11			
Exophiala pisciphila McGinnis & Ajello	0.15		0.11			
Exophiala xenobiotica de Hoog, J.S. Zeng, Harrak & Deanna A. Sutton						
Filobasidium magnum (Lodder & Kreger-van Rij) Xin Zhan Liu, F.Y. Bai, M. Groenew. & Boekhout		0.26				
Furcasterigmium furcatum (Gams) Giraldo López & Crous			0.11			
Fusarium brachygibbosum Padwick			0.11			
Fusarium cerealis (Cooke) Sacc.				1.03		
Fusarium incarnatum/equiseti sp. complex				2.91		0.59
Fusarium cortaderiae O'Donnell, T. Aoki, Kistler & Geiser			0.23			
***Fusarium culmorum* (Wm.G. Sm.) Sacc.**		0.33	0.11	1.79		
Fusarium equiseti (Corda) Sacc.				0.17		
Fusarium flocciferum Corda				0.26		
Fusarium incarnatum (Roberge ex Desm.) Sacc.				0.34		0.18
***Fusarium oxysporum* Schltdl**.		0.20	0.11			
Geotrichum candidum Link		0.07		0.17		
***Gibellulopsis nigrescens* (Pethybr.) Zare, W. Gams & Summerb.**			0.45	0.07		
Gliomastix murorum (Corda) S. Hughes			0.23		4.15	
Halosphaeriaceae sp.					0.45	
Hongkongmyces thailandica Phukhams. & K.D. Hyde	0.15		0.57			
Hypocreales sp.					0.05	
Kiflimonium curvulum (W. Gams) Summerb., J.A. Scott, Guarro & Crous			0.11			
Lasionectria marigotensis Lechat & J. Fourn.			0.23			
Lecanicillium fusisporum (W. Gams) Zare & W. Gams					0.45	
Lentitheciaceae sp.			0.11			

Malbranchea circinata Sigler & J.W. Carmich.	0.44				0.45	
Marquandomyces marquandii (Massee) Samson, Houbraken & Luangsa-ard						0.71
Massarina rubi (Fuckel) Sacc.					2.71	
Massarina sp.1			1.13			
Massarina sp.2	0.12					
Massarina sp.3	0.30					
Metarhizium brunneum Petch			0.11			
Microascus paisii (Pollacci) Sand.-Den., Gené & Guarro	1.32		0.11			
Microascus sp.					0.45	
Microdiplodia sp.					1.80	
Microdochium seminicola M. Hern.-Restr., Seifert, Clear & B. Dorn			0.11			
Mortierella alpina Peyronel		0.40		2.14		
Mucor circinelloides Tiegh.		0.07				
Mucor heterogamus Vuill.		0.26				
Mucor hiemalis Wehmer				1.20		
Mucor plumbeus Bonord.				1.11		0.06
Myrmecridium phragmitis Crous			0.23			
Myrmecridium schulzeri (Sacc.) Arzanlou, W. Gams & Crous			0.11			
Myrothecium sp.					0.90	
Nectria inventa Pethybr.			0.11			
Nectria pseudotrichia Berk. & M.A. Curtis						0.06
Neocosmospora solani (Mart.) L. Lombard & Crous	0.15		0.68			
Neopestalotiopsis clavispora (G.F. Atk.) Maharachch., K.D. Hyde & Crous				0.26		
Niesslia tenuis (W. Gams) W. Gams			25.03			
Nothophoma quercina (Syd. & P. Syd.) Qian Chen & L. Cai		0.13				
Papiliotrema laurentii (Kuff.) Xin Zhan Liu, F.Y. Bai, M. Groenew. & Boekhout				0.34		
Paraconiothyrium cyclothyrioides Verkley					0.05	
Parasarocladium debruynii L. Lombard	0.44					
Parasarocladium radiatum (Sukapure & Thirum.) Summerb., J.A. Scott, Guarro & Crous	1.61		0.11			
Parasarocladium wereldwijsianum Hern.-Restr.	0.59		0.34			
Parathielavia kuwaitensis (Moustafa) X. Wei Wang & Houbraken			0.91			
Parathyridaria flabelliae E. Bovio, A. Poli, Prigione & Varese			0.11			
Parengyodontium album (Limber) C.C. Tsang, J.F.W. Chan, W.M. Pong, J.H.K. Chen, A.H.Y. Ngan, M. Cheung, C.K.C. Lai, D.N.C. Tsang, S.K.P. Lau & P.C.Y. Woo		0.40				
Penicillium jacksonii K.G. Rivera, Houbraken & Seifert				1.03		
***Penicillium antarcticum* A.D. Hocking & C.F. McRae**	0.29				0.90	0.06
***Penicillium atramentosum* Thom**	0.29	0.20		1.20		
***Penicillium brevicompactum* Dierckx**		0.13	0.79	2.56	3.16	0.18
Penicillium canescens Sopp				3.85		0.06
Penicillium canis S.W. Peterson	0.15					
***Penicillium chrysogenum* Thom**			20.05	0.09		
Penicillium cinereoatrum Chalab.					0.90	
Penicillium citreonigrum Dierckx			0.23			
Penicillium coprophilum (Berk. & M.A. Curtis) Seifert & Samson			0.34			
Penicillium coralligerum Nicot & Pionnat				0.09		

***Penicillium crustosum* Thom**	0.59	0.59		1.03		38.48
Penicillium cyclopium Westling		0.86				
Penicillium daleae K.W. Zaleski		0.79		0.60		
***Penicillium echinulatum* Raper & Thom ex Fassat.**		5.55	0.23	0.09	0.90	0.94
Penicillium egyptiacum J.F.H. Beyma				2.39		0.12
Penicillium expansum Link		0.13		1.11		0.65
Penicillium fellutanum Biourge	2.34		0.11			
Penicillium freii Frisvad & Samson						0.12
***Penicillium glabrum* (Wehmer) Westling**	0.88	0.07	0.34	2.48		0.53
Penicillium glandicola (Oudem.) Seifert & Samson		0.73		18.89		
Penicillium griseofulvum Dierckx		0.59		2.56		0.24
Penicillium canescens Sopp		0.13				
Penicillium herquei Bainier & Sartory						0.12
Penicillium hoeksii Houbraken		2,44		0.43		0.06
Penicillium hordei Stolk				6.92		0.29
Penicillium jacksonii K.G. Rivera, Houbraken & Seifert		0.07				0.06
Penicillium janczewskii K.W. Zaleski	2.64		0.34		2.25	
Penicillium javanicum J.F.H. Beyma	0.59					
Penicillium manginii Duché & R. Heim						11.96
Penicillium melanoconidium (Frisvad) Frisvad & Samson		0.66				
Penicillium menonorum S.W. Peterson	0.15					
Penicillium murcianum C. Ramírez & A.T. Martínez	1.46		0.91		4.10	
Penicillium nalgiovense Laxa				0.09		
Penicillium olsonii Bainier & Sartory				0.26		0.06
Penicillium osmophilum Stolk & Veenb.-Rijks		45.18				
Penicillium oxalicum Currie & Thom	1.17		0.23			
Penicillium pancosmium Houbraken, Frisvad & Samson						0.06
Penicillium paneum Frisvad	0.29					
Penicillium parvulum S.W. Peterson & B.W. Horn	0.59					
Penicillium restrictum J.C. Gilman & E.V. Abbott	0.15		0.11		2.25	
Penicillium roseopurpureum Dierckx			0.91			
Penicillium sacculum E. Dale			1.25			
Penicillium scabrosum Frisvad, Samson & Stolk				0.09		
***Penicillium simplicissimum* (Oudem.) Thom**	8.93	0.01				
***Penicillium sizovae* Baghd.**			1.81	0.17	2.71	
***Penicillium solitum* Westling**		0.07			0.05	
Penicillium sp1	0.15					
Penicillium sp2			0.34			
Penicillium spathulatum Frisvad & Samson						0.06
Penicillium steckii K.W. Zaleski						0.06
Penicillium yezoense Hanzawa						0.18
Penicillium ubiquetum Houbraken, Frisvad & Samson				0.17		0.12
Penicillium vancouverense Houbraken, Frisvad & Samson						0.06
Penicillium venetum (Frisvad) Frisvad		0.07		0.60		

Penicillium viridicatum Westling		0.07		0.94		0.18
Penicillium waksmanii K.W. Zaleski			0.23			
***Penicillium yarmokense* Baghd.**		0.07		0.09	0.90	
Periconia byssoides Pers.					0.45	
Phaeosphaeria sp.					0.45	
Phaeosphaeriaceae sp.	0.15					
Pholiota gummosa (Lasch) Singer	0.15					
Phoma leveillei Boerema & G.J. Bollen			0.91			
Pichia methanolica Makig.			0.11			
Pichia occidentalis (Kurtzman, M.J. Smiley & C.J. Johnson) Kurtzman, Robnett & Basehoar-Powers	0.44					
Plectosphaerella cucumerina (Lindf.) W. Gams			0.79			
Pleosporales sp.1 (C34)	0.15					
Pleosporales sp.2(c46)	0.07					
Preussia sp.	0.15					
Priceomyces carsonii (Phaff & E.P. Knapp) M. Suzuki			0.23			
Pseudeurotium bakeri C. Booth	0.15					
Pseudeurotium ovale Stolk	0.29		0.11		0.05	
Pseudogymnoascus pannorum (Link) Minnis & D.L. Lindner						0.06
Purpureocillium lilacinum (Thom) Luangsa-ard, Houbraken, Hywel-Jones & Samson			2.38		5.41	
Pyrenochaetopsis tabarestanensis M. Papizadeh, Soudi, Amini, Wijayaw. & K.D. Hyde	0.15					
Rhizopus orzyae Went & Prins. Geerl.		0.26		0.26		
Rhodotorula diobovata (S.Y. Newell & I.L. Hunter) Q.M. Wang, F.Y. Bai, M. Groenew. & Boekhout		23.84		21.71		0.53
Rhodotorula mucilaginosa (A. Jörg.) F.C. Harrison		1.19				0.12
Rhodotorula sphaerocarpa (S.Y. Newell & Fell) Q.M. Wang, F.Y. Bai, M. Groenew. & Boekhout		0.07				
Roussoella sp.			0.23		2.25	
Roussoellaceae sp.					0.45	
Sarocladium kiliense (Grütz) Summerbell	0.29					
Scedosporium dehoogii Gilgado, Cano, Gené & Guarro	2.00		0.79			
Scytalidium lignicola Pesante			0.11			
Solicoccozyma aeria (Saito) Yurkov				0.09		0.06
Sordariomycetes sp.					0.45	
Sporothrix inflata de Hoog	1.02					
Sporothrix nigrograna (Masuya) Z.W. de Beer, T.A. Duong & M.J. Wingf.	0.15					
Stachybotryna excentrica Gené, M. Calduch, Abdullah & Guarro			0.11		0.90	
***Stachybotrys chartarum* (Ehrenb.) S. Hughes**	0.15					0.12
Stachybotrys chlorohalonata B. Andersen & Thrane	6.73		0.34		0.45	
Stachylidium bicolor Link	0.29		0.11			
Stagonosporopsis cucurbitacearum (Fr.) Aveskamp, Gruyter & Verkley		0.40				
Stilbella fimetaria (Pers.) Lindau			0.11			
Stilbella sp.					0.45	
Suhomyces ambrosiae (Kurtzman) M. Blackw. & Kurtzman						0.06
Suhomyces sp.						0.06

Symmetrospora coprosmae (Hamamoto & Nakase) Q.M. Wang, F.Y. Bai, M. Groenew. & Boekhout	0.15		0.34			
Talaromyces albobiverticillius (H.M. Hsieh, Y.M. Ju & S.Y. Hsieh) Samson, Yilmaz, Frisvad & Seifert					0.45	
Talaromyces allahbadensis (B.S. Mehrotra & D. Kumar) Samson, N. Yilmaz & Frisvad			0.11			
Talaromyces argentinensis Jurjević & S.W. Peterson					0.45	
Talaromyces borbonicus Houbraken					1.35	
Talaromyces catalonicus Guevara-Suarez, Gené & Guarro			0.34			
Talaromyces flavus (Klöcker) Stolk & Samson			0.23			
Talaromyces minioluteus (Dierckx) Samson, Yilmaz, Frisvad & Seifert	0.15				1.80	
Talaromyces muroii Yaguchi, Someya & Udagawa	0.15					
Talaromyces verruculosus (Peyronel) Samson, Yilmaz, Frisvad & Seifert			0.11			
Talaromyces versatilis P.F. Cannon, Bridge & Buddie	0.15		0.68		0.45	
Talaromyces wortmannii (Klöcker) C.R. Benj.	1.90		0.11		0.05	
Thelebolus sp. 1	0.15					
Thelebolus sp. 2		0.07				
Thelonectria veuillotiana (Sacc. & Roum.) P. Chaverri & C. Salgado						0.06
Tilachlidium brachiatum (Batsch) Petch	0.15					
Tilletiopsis washingtonensis Nyland			0.11		2.25	
Trematosphaeria grisea (J.E. Mackinnon, Ferrada & Montemart.) S.A. Ahmed, Sande, Fahal & de Hoog	1.90					
Trichoderma afroharzianum (Batsch) Petch			0.68			
Trichoderma alni Jaklitsch					0.45	
Trichoderma atrobrunneum F.B. Rocha, P. Chaverri & Jaklitsch				0.09		
Trichoderma atroviride P. Karst.		2.84	0.57	0.94		0.59
Trichoderma caerulescens (Jaklitsch & Voglmayr) Jaklitsch & Voglmayr					0.45	
Trichoderma ceratophylli Z.F. Yu & X. Du			0.11			
***Trichoderma gamsii* Samuels & Druzhin.**			0.23	2.05		
Trichoderma hamatum (Bonord.) Bainier			0.11			
***Trichoderma harzianum* Rifai**	24.01	4.23	3.28	7.01	2.39	5.54
Trichoderma koningiopsis Samuels, C. Suárez & H.C. Evans			0.11			
***Trichoderma longibrachiatum* Rifai**		1.65	0.11			
Trichoderma orientale (Samuels & Petrini) Jaklitsch & Samuels		0.20		0.17		
Trichoderma paraviridescens Jaklitsch, Samuels & Voglmayr				0.09		
Trichoderma sinuosum P. Chaverri & Samuels					0.99	
Trichoderma sp. 1			0.57			
Trichoderma sp. 2			0.79			
Trichoderma sp. 3						0.06
Trichoderma sp.4						0.06
Trichoderma tomentosum Bissett			0.23			
Trichoderma velutinum Bissett, C.P. Kubicek & Szakacs			0.34		0.90	
Trichoderma viridescens (A.S. Horne & H.S. Will.) Jaklitsch & Samuels					3.16	
***Trichosporiella flavificans* (Nakase) de Hoog, Rodr. Mir. & Oorschot**	0.13	0.06				
Truncatella angustata (Pers.) S. Hughes				0.16		
Ustilago sp.	0.13					
Valsaria spartii Maubl.			0.11			

Vanrija albida (C. Ramírez) M. Weiß				0.10	
Vishniacozyma heimaeyensis Vishniac ex Xin Zhan Liu, F.Y. Bai, M. Groenew. & Boekhout		0.20			
Volutella ciliata (Alb. & Schwein.) Fr.			0.91		
Wardomycopsis humicola (G.L. Barron) Udagawa & Furuya	0.59		1.02		5.41
Westerdykella centenaria Crous, van Diepeningen & A.-C. Normand					0.90
Westerdykella dispersa (Clum) Cejp & Milko	0.88				
