# Supplementary material for: The Culturable Mycobiota of Sediments and Associated Microplastics: From a Harbor to a Marine Protected Area, a Comparative Study
Source: J Fungi (Basel). 2022 Aug 31;8(9):927. doi: 10.3390/jof8090927 (PMC9501098; doi:10.3390/jof8090927)
Supplement: Supplementary file 1 [file jof-08-00927-s001.zip › Table S7.pdf]

Table S7. Fungal taxa isolated from Solid sediment (SS), Watery sediment (WS) and microplastics (MPs) and recovered from other marine environments.

Taxa	SS	WS	MPs	Marine environments
Acremonium antarcticum (Spegazzini) D. Hawksworth	x			FR
Acremonium pilosum Giraldo, Guarro, Cano & Gene	x			FR
Acremonium sclerotigenum (Moreau & R. Moreau ex Valenta) W. Gams	x			[1,2]
Acremonium tubakii W. Gams	x			[3,4]
Acrostalagmus luteoalbus (Link) Zare, W. Gams & Schroers	x	x		[5,6]
Albifimbria verrucaria (Alb. & Schwein.) L. Lombard & Crous		x		FR
Alternaria alternata (Fr.) Keissl.	x	x		[5,7]
Alternaria sp.		x		-
Amesia nigricolor (L.M. Ames) X. Wei Wang & Samson	x			[8]
Annulohypoxylon multiforme (Fr.) Y.M. Ju, J.D. Rogers & H.M. Hsieh	x			FR
Apiospora arundinis (Corda) Pintos & P. Alvarado		x		[9,10]
Apiospora marii (Larrondo & Calvo) Pintos & P. Alvarado		x		[11]
Apiospora stipae Crous & R.K. Schumach.	x			FR
Apiotrichum dulcitum (Berkhout) Yurkov & Boekhout		x		[12]
Apiotrichum laibachii (Windisch) Yurkov & Boekhout		x		FR
Armillaria mellea (Vahl) P. Kumm.	x			FR
Arthopyrenia salicis A. Massal.	x			FR
Arthrinium arundinis (Corda) Dyko & B. Sutton	x			[9,13,14]
Arthrinium kogelbergense Crous		x		FR
Arthrinium marii Larrondo & Calvo	x			[11]
Arthrinium phaeospermum (Corda) M.B. Ellis	x	x		[14]
Ascochyta sp.		x		-
Ascomycota sp.1	x			-
Ascomycota sp.2	x			-
Ascomycota sp.3	x			-
Ascomycota sp.4	x			-
Ascomycota sp.5	x			-
Aspergillus aureolatus Munt.-Cvetk. & Bata	x			[15,16]
Aspergillus domesticus Sklenar, Houbraken, Zalar & Hubka			x	FR
Aspergillus europaeus Hubka, A. Nováková, Samson, Houbraken, Frisvad, M. Kolařík		x		[17,18]
Aspergillus flavipes (Bainier & Sartory) Thom & Church	x			[19]
Aspergillus fumigatus Fresen.	x			[4,5,11]
Aspergillus germanicus Frisvad, Varga & Samson	x			FR
Aspergillus heyangensis Z.T. Qi, Z.M. Sun & Yu X. Wang	x	x		[11]
Aspergillus insuetus (Bainier) Thom & Church	x	x		[5,20]
Aspergillus jensenii Jurjević, S.W. Peterson & B.W. Horn	x		x	[21]
Aspergillus niger Tiegh.	x			[5,22,23]
Aspergillus pseudodeflectus Samson & Mouch.	x			[5,24,25]
Aspergillus pseudoglaucus Blochwitz	x			[9,26]
Aspergillus sp.	x			-
Aspergillus spelaeus A. Nováková, Hubka, M. Kolařík & S.W. Peterson		x		[27]
Aspergillus tabacinus Nakaz., Y. Takeda, Simo & A. Watan.	x			[26]
Aspergillus templicola Visagie, Hirooka & Samson	x			FR
Aspergillus terreus Thom	x			[5]

Aspergillus tubingensis Mosseray		x		[5]
Aspergillus ustus (Bainier) Thom & Church	x			[5]
Aspergillus versicolor (Vuill.) Tirab.	x			[5]
Aureobasidium pullulans (de Bary) G. Arnaud	x	x	x	[5,13]
Auxarthron conjugatum (Kuehn) G.F. Orr & Kuehn	x			FR
Auxarthron thaxteri (Kuehn) G.F. Orr & Kuehn	x			FR
Beauveria bassiana (Bals.-Criv.) Vuill.	x			[5]
Beauveria brongniartii (Sacc.) Petch	x			[5]
Beauveria felina (DC.) J.W. Carmich.	x			[5,28]
Beauveria varroae S.A. Rehner & R.A. Humber	x			FR
Biatriospora sp. (MUT 4883)	x			-
Bionectriaceae sp	x			-
Bjerkandera adusta (Willd.) P. Karst.	x		x	[29]
Brunneomyces brunnescens (W. Gams) Giraldo, Gene & Guarro	x			FR
Cadophora luteo-olivacea (J.F.H. Beyma) T.C. Harr. & McNew	x			[30]
Candida membranifaciens (Lodder & Kreger) Wick. & K.A. Burton	x			[5,31,32]
Candida norvegica (Reiersöl) S.A. Mey. & Yarrow	x	x		[5,32]
Candida pseudolambica M.T. Sm. & Poot	x			[5,33]
Candida sp.	x			-
Cephalotrichum asperulum (J.E. Wright & S. Marchand) Sand.-Den., Guarro & Gené		x		FR
Cephalotrichum columnare (H.J. Swart) S.P. Abbott	x	x		FR
Cephalotrichum gorgonifer (Bainier) Sand.-Den., Gené & Guarro	x			[5]
Cephalotrichum inflatum Y.L. Jiang & T.Y. Zhang		x		-
Cephalotrichum stemonitis (Pers.) Nees	x			[34]
Chaetomium cochliodes Palliser		x		FR
Chaetomium globosum Kunze	x			[5,35]
Chrysosporium lobatum Scharapov	x			[9,36]
Cistella sp.	x			-
Cladosporium aggregatocicatricatum Bensch, Crous & U. Braun	x	x		[29]
Cladosporium allicinum (Fr.) Bensch, U. Braun & Crous	x	x		[13,37]
Cladosporium asperulatum Bensch, Crous & U. Braun	x			FR
Cladosporium cladosporioides (Fresen.) G.A. de Vries	x	x	x	[5,14,34]
Cladosporium halotolerans Zalar, de Hoog & Gunde-Cim.	x	x	x	[11,14,38]
Cladosporium inversicolor Bensch, Crous & U. Braun		x		FR
Cladosporium limoniforme Bensch, Crous & U. Braun	x	x		[39]
Cladosporium perangustum Bensch, Crous & U. Braun	x	x		[40]
Cladosporium pseudocladosporioides Bensch, Crous & U. Braun	x	x	x	[14,37]
Cladosporium psychrotolerans Zalar, de Hoog & Gunde-Cim.	x	x		[41]
Cladosporium ramotenellum K. Schub., Zalar, Crous & U. Braun		x	x	[42]
Cladosporium sp.2	x			-
Cladosporium sp1	x			-
Cladosporium sphaerospermum Penz		x		[5,43]
Cladosporium tenellum K. Schub., Zalar, Crous & U. Braun		x		FR
Cladosporium velox Zalar, de Hoog & Gunde-Cim.		x		FR
Cladosporium westerdijkiae Bensch & Samson		x		FR
Cladosporium xylophilum Bensch, Shabunin, Crous & U. Braun		x		[11]
Clonostachys rosea (Link) Schroers, Samuels, Seifert & W. Gams	x	x		[44]
Clonostachys rossmaniae Schroers	x			FR
Clonostachys sp.	x			-

Coniochaeta sp.	x			-
Cyberlindnera saturnus (Klöcker) Minter	x			[5]
Cystobasidium slooffiae (E.K. Novák & Vörös-Felkai) Yurkov, Kachalkin, H.M. Daniel, M. Groenew., Libkind, V. de Garcia, Zalar, Gouliam., Boekhout & Begerow			x	[45]
Debaryomyces hansenii (Zopf) Lodder & Kreger	x	x		[5]
Debaryomyces marasmus Di Menna	x			FR
Diaporthe columnaris (D.F. Farr & Castl.) Udayanga & Castl.	x			FR
Dichotomopilus funicola (Cooke) X. Wei Wang & Samson	x			[5]
Discula destructiva Redlin	x			FR
Doratomyces microsporus (Sacc.) F.J. Morton & G. Sm.		x		FR
Emericella pluriseminata Stchigel & Guarro	x			FR
Emericellopsis maritima Beliakova	x			[5,38]
Emericellopsis minima Stolk	x			[5]
Epicoccum nigrum Link	x			[14,38]
Eupenicillium crustaceum F. Ludw.	x			FR
Exophiala equina (Pollacci) de Hoog, Vicente, Najafzadeh, Harrak, Badali & Seyedmousavi	x			FR
Exophiala pisciphila McGinnis & Ajello	x			[5]
Exophiala xenobiotica de Hoog, J.S. Zeng, Harrak & Deanna A. Sutton	x			[5]
Filobasidium magnum (Lodder & Kreger-van Rij) Xin Zhan Liu, F.Y. Bai, M. Groenew. & Boekhout		x		[46]
Furcasterigmium furcatum (Gams) Giraldo López & Crous	x			[5]
Fusarium brachygibbosum Padwick	x			[14]
Fusarium cerealis (Cooke) Sacc.		x		FR
Fusarium incarnatum/equiseti sp. complex		x		[5]
Fusarium cortaderiae O'Donnell, T. Aoki, Kistler & Geiser	x			FR
Fusarium culmorum (Wm.G. Sm.) Sacc.	x	x		[5]
Fusarium equiseti (Corda) Sacc.		x		[5]
Fusarium flocciferum Corda		x		FR
Fusarium incarnatum (Roberge ex Desm.) Sacc.		x		[5]
Fusarium oxysporum Schltdl.	x	x		[47,48]
Geotrichum candidum Link		x		[49]
Gibellulopsis nigrescens (Pethybr.) Zare, W. Gams & Summerb.	x	x		[9]
Gliomastix murorum (Corda) S. Hughes	x			[5]
Halosphaeriaceae sp.	x			-
Hongkongmyces thailandica Phukhams. & K.D. Hyde	x			FR
Hypocreales sp.	x			-
Kiflimonium curvulum (W. Gams) Summerb., J.A. Scott, Guarro & Crous	x			FR
Kondoa aeria Á. Fonseca, J.P. Samp. & Fell			x	[50–52]
Lasionectria marigotensis Lechat & J. Fourn.	x			FR
Lecanicillium fusisporum (W. Gams) Zare & W. Gams	x			[5]
Lentitheciaceae sp.	x			-
Malbranchea circinata Sigler & J.W. Carmich.	x			FR
Marquandomyces marquandii (Massee) Samson, Houbraken & Luangsa-ard		x		[53]
Massarina rubi (Fuckel) Sacc.	x			[54]
Massarina sp.1	x			-
Massarina sp.2	x			-
Massarina sp.3	x			-
Metarhizium brunneum Petch	x			FR

Microascus paisii (Pollacci) Sand.-Den., Gené & Guarro	x			FR
Microascus sp.	x			-
Microdiplodia sp.	x			-
Microdochium seminicola M. Hern.-Restr., Seifert, Clear & B. Dorn	x			FR
Mortierella alpina Peyronel		x		FR
Mucor circinelloides Tiegh.		x		[55]
Mucor heterogamus Vuill.		x		FR
Mucor hiemalis Wehmer		x		FR
Mucor plumbeus Bonord.		x		FR
Myrmecridium phragmitis Crous	x			FR
Myrmecridium schulzeri (Sacc.) Arzanlou, W. Gams & Crous	x			[5]
Myrothecium sp.	x			-
Nectria inventa Pethybr.	x			[56]
Nectria pseudotrichia Berk. & M.A. Curtis		x		-
Neocosmospora solani (Mart.) L. Lombard & Crous	x			[57]
Neopestalotiopsis clavispora (G.F. Atk.) Maharachch., K.D. Hyde & Crous		x		FR
Niesslia tenuis (W. Gams) W. Gams	x			FR
Nothophoma quercina (Syd. & P. Syd.) Qian Chen & L. Cai		x		FR
Papiliotrema laurentii (Kuff.) Xin Zhan Liu, F.Y. Bai, M. Groenew. & Boekhout		x		FR
Paraconiothyrium cyclothyrioides Verkley	x			[5]
Parasarocladium debruynii L. Lombard	x			FR
Parasarocladium radiatum (Sukapure & Thirum.) Summerb., J.A. Scott, Guarro & Crous	x			FR
Parasarocladium wereldwijsianum Hern.-Restr.	x			FR
Parathielavia kuwaitensis (Moustafa) X. Wei Wang & Houbraken	x			[58]
Parathyridaria flabelliae E. Bovio, A. Poli, Prigione & Varese	x			[59]
Parengyodontium album (Limber) C.C. Tsang, J.F.W. Chan, W.M. Pong, J.H.K. Chen, A.H.Y. Ngan, M. Cheung, C.K.C. Lai, D.N.C. Tsang, S.K.P. Lau & P.C.Y. Woo		x	x	[5,60,61]
Penicillium jacksonii K.G. Rivera, Houbraken & Seifert		x		[5]
Penicillium antarcticum A.D. Hocking & C.F. McRae	x	x		[13,21,37,62]
Penicillium atramentosum Thom	x	x		[47]
Penicillium bialowiezense K.W. Zaleski			x	[63]
Penicillium brevicompactum Dierckx	x	x	x	[4,5,13]
Penicillium canescens Sopp		x		[5,14]
Penicillium canis S.W. Peterson	x			FR
Penicillium chrysogenum Thom	x	x		[5,14]
Penicillium cinereoatrum Chalab.	x			[64]
Penicillium citreonigrum Dierckx	x			[65]
Penicillium coprophilum (Berk. & M.A. Curtis) Seifert & Samson	x			[65]
Penicillium coralligerum Nicot & Pionnat		x		[66]
Penicillium crustosum Thom	x	x		[67]
Penicillium cyclopium Westling		x		[68]
Penicillium daleae K.W. Zaleski		x		FR
Penicillium echinulatum Raper & Thom ex Fassat.	x	x		[69]
Penicillium egyptiacum J.F.H. Beyma		x		FR
Penicillium expansum Link		x		[5]
Penicillium fellutanum Biourge	x			[70]
Penicillium freii Frisvad & Samson		x		FR

Penicillium glabrum (Wehmer) Westling	x	x		[5,64]
Penicillium glandicola (Oudem.) Seifert & Samson		x		FR
Penicillium griseofulvum Dierckx		x	x	[71]
Penicillium canescens Sopp		x		[5,14]
Penicillium herquei Bainier & Sartory		x		[72]
Penicillium hoeksii Houbraken		x		FR
Penicillium hordei Stolk		x		FR
Penicillium jacksonii K.G. Rivera, Houbraken & Seifert		x		FR
Penicillium janczewskii K.W. Zaleski	x			[5]
Penicillium javanicum J.F.H. Beyma	x			[73]
Penicillium manginii Duché & R. Heim		x		FR
Penicillium melanoconidium (Frisvad) Frisvad & Samson		x		FR
Penicillium menonorum S.W. Peterson	x			FR
Penicillium murcianum C. Ramírez & A.T. Martínez	x			[64]
Penicillium nalgiovense Laxa		x		FR
Penicillium olsonii Bainier & Sartory		x		FR
Penicillium osmophilum Stolk & Veenb.-Rijks		x		FR
Penicillium oxalicum Currie & Thom	x			[5]
Penicillium pancosmium Houbraken, Frisvad & Samson		x		FR
Penicillium paneum Frisvad	x			[5]
Penicillium parvulum S.W. Peterson & B.W. Horn	x			FR
Penicillium restrictum J.C. Gilman & E.V. Abbott	x			[5]
Penicillium roseopurpureum Dierckx	x			[9]
Penicillium sacculum E. Dale	x			[5]
Penicillium scabrosum Frisvad, Samson & Stolk		x		FR
Penicillium simplicissimum (Oudem.) Thom	x	x		[5]
Penicillium sizovae Baghd.	x	x		[74]
Penicillium solitum Westling	x	x		[5]
Penicillium sp.1	x			-
Penicillium sp.2	x			-
Penicillium spathulatum Frisvad & Samson		x		FR
Penicillium steckii K.W. Zaleski		x		[64]
Penicillium yezoense Hanzawa		x		FR
Penicillium ubiquetum Houbraken, Frisvad & Samson		x		[75]
Penicillium vancouverense Houbraken, Frisvad & Samson		x		FR
Penicillium venetum (Frisvad) Frisvad		x		FR
Penicillium viridicatum Westling		x		FR
Penicillium waksmanii K.W. Zaleski	x			[5]
Penicillium yarmokense Baghd.	x	x		FR
Periconia byssoides Pers.	x			[5]
Phaeosphaeria sp.	x			-
Phaeosphaeriaceae sp.	x			-
Pholiota gummosa (Lasch) Singer	x			FR
Phoma leveillei Boerema & G.J. Bollen	x			[76]
Pichia methanolica Makig.	x			FR
Pichia occidentalis (Kurtzman, M.J. Smiley & C.J. Johnson) Kurtzman, Robnett & Basehoar-Powers	x			[5]
Plectosphaerella cucumerina (Lindf.) W. Gams	x			[77]
Pleosporales sp.1	x			-
Pleosporales sp.2	x			-

Preussia sp.	x			-
Priceomyces carsonii (Phaff & E.P. Knapp) M. Suzuki	x			[5]
Pseudeurotium bakeri C. Booth	x			[21]
Pseudeurotium ovale Stolk	x			FR
Pseudogymnoascus pannorum (Link) Minnis & D.L. Lindner		x		[5]
Purpureocillium lilacinum (Thom) Luangsa-ard, Houbraken, Hywel-Jones & Samson	x			[5]
Pyrenochaetopsis tabarestanensis M. Papizadeh, Soudi, Amini, Wijayaw. & K.D. Hyde	x			FR
Rhizopus orzyae Went & Prins. Geerl.		x		[78,79]
Rhodotorula diobovata (S.Y. Newell & I.L. Hunter) Q.M. Wang, F.Y. Bai, M. Groenew. & Boekhout		x		[80–83]
Rhodotorula mucilaginosa (A. Jörg.) F.C. Harrison		x		[5,82]
Rhodotorula sphaerocarpa (S.Y. Newell & Fell) Q.M. Wang, F.Y. Bai, M. Groenew. & Boekhout		x		[82]
Roussoella sp.	x			-
Roussoellaceae sp.	x			-
Sakaguchia dacryoidea (Fell, I.L. Hunter & Tallman) Y. Yamada, K. Maeda & Mikata			x	[84–87]
Sarocladium kiliense (Grütz) Summerbell	x			[88]
Scedosporium dehoogii Gilgado, Cano, Gené & Guarro	x			[89]
Scytalidium lignicola Pesante	x			[90]
Sesquicillium microsporum (Jaap) Veenb.-Rijks & W. Gams			x	[91]
Solicoccozyma aeria (Saito) Yurkov		x		FR
Sordariomycetes sp.	x			-
Sporothrix inflata de Hoog	x			[11]
Sporothrix nigrograna (Masuya) Z.W. de Beer, T.A. Duong & M.J. Wingf.	x			FR
Stachybotryna excentrica Gené, M. Calduch, Abdullah & Guarro	x			FR
Stachybotrys chartarum (Ehrenb.) S. Hughes	x	x		[5]
Stachybotrys chlorohalonata B. Andersen & Thrane	x			[5]
Stachylidium bicolor Link	x			[5]
Stagonosporopsis cucurbitacearum (Fr.) Aveskamp, Gruyter & Verkley		x		[38]
Stilbella fimetaria (Pers.) Lindau	x			FR
Stilbella sp.	x			-
Suhomyces ambrosiae (Kurtzman) M. Blackw. & Kurtzman		x		FR
Suhomyces sp.		x		-
Symmetrospora coprosmae (Hamamoto & Nakase) Q.M. Wang, F.Y. Bai, M. Groenew. & Boekhout	x			FR
Talaromyces albobiverticillius (H.M. Hsieh, Y.M. Ju & S.Y. Hsieh) Samson, Yilmaz, Frisvad & Seifert	x			[92]
Talaromyces allahbadensis (B.S. Mehrotra & D. Kumar) Samson, N. Yilmaz & Frisvad	x			FR
Talaromyces argentinensis Jurjević & S.W. Peterson	x			FR
Talaromyces borbonicus Houbraken	x			FR
Talaromyces catalonicus Guevara-Suarez, Gené & Guarro	x			FR
Talaromyces flavus (Klöcker) Stolk & Samson	x			[5]
Talaromyces minioluteus (Dierckx) Samson, Yilmaz, Frisvad & Seifert	x			[5]
Talaromyces muroii Yaguchi, Someya & Udagawa	x			FR
Talaromyces verruculosus (Peyronel) Samson, Yilmaz, Frisvad & Seifert	x			[5]
Talaromyces versatilis P.F. Cannon, Bridge & Buddie	x			[93]
Talaromyces wortmannii (Klöcker) C.R. Benj.	x			FR

Thelebolus sp.1	x			-
Thelebolus sp. 2		x		-
Thelonectria veuillotiana (Sacc. & Roum.) P. Chaverri & C. Salgado		x		FR
Tilachlidium brachiatum (Batsch) Petch	x			[19]
Tilletiopsis washingtonensis Nyland	x			FR
Trematosphaeria grisea (J.E. Mackinnon, Ferrada & Montemart.) S.A. Ahmed, Sande, Fahal & de Hoog	x			FR
Trichoderma afroharzianum (Batsch) Petch	x			[94]
Trichoderma alni Jaklitsch	x			FR
Trichoderma atrobrunneum F.B. Rocha, P. Chaverri & Jaklitsch		x		FR
Trichoderma atroviride P. Karst.	x	x		[5,64]
Trichoderma caerulescens (Jaklitsch & Voglmayr) Jaklitsch & Voglmayr	x			FR
Trichoderma ceratophylli Z.F. Yu & X. Du	x			FR
Trichoderma gamsii Samuels & Druzhin.	x	x		[94]
*Trichoderma hamatum (*Bonord.) Bainier	x			[5]
Trichoderma harzianum Rifai	x	x		[5]
Trichoderma koningiopsis Samuels, C. Suárez & H.C. Evans	x			FR
Trichoderma longibrachiatum Rifai	x	x		[5]
Trichoderma orientale (Samuels & Petrini) Jaklitsch & Samuels		x		[94]
Trichoderma paraviridescens Jaklitsch, Samuels & Voglmayr		x		[94]
Trichoderma sinuosum P. Chaverri & Samuels	x			FR
Trichoderma sp. 1	x			-
Trichoderma sp. 2	x			-
Trichoderma sp. 3		x		-
Trichoderma sp.4		x		-
Trichoderma tomentosum Bissett	x			FR
Trichoderma velutinum Bissett, C.P. Kubicek & Szakacs	x			FR
Trichoderma viridescens (A.S. Horne & H.S. Will.) Jaklitsch & Samuels	x			FR
Trichosporiella flavificans (Nakase) de Hoog, Rodr. Mir. & Oorschot	x	x		[73]
Truncatella angustata (Pers.) S. Hughes		x		[95]
Ustilago sp.	x			-
Valsaria spartii Maubl.	x			FR
Vanrija albida (C. Ramírez) M. Weiß		x		FR
Vishniacozyma carnescens (Verona & Luchetti) Xin Zhan Liu, F.Y. Bai, M. Groenew. & Boekhout			x	FR
Vishniacozyma heimaeyensis Vishniac ex Xin Zhan Liu, F.Y. Bai, M. Groenew. & Boekhout		x		FR
Volutella ciliata (Alb. & Schwein.) Fr.	x			[19]
Wardomycopsis humicola (G.L. Barron) Udagawa & Furuya	x			[55]
Westerdykella centenaria Crous, van Diepeningen & A.-C. Normand	x			FR
Westerdykella dispersa (Clum) Cejp & Milko	x			[96]

*FR First record

References

[1] J.M. Wong Chin, D. Puchooa, T. Bahorun, R. Jeewon, Molecular characterization of marine fungi associated with Haliclona sp. (sponge) and Turbinaria conoides and Sargassum portierianum (brown algae), Proceedings of the National Academy of Sciences India Section B - Biological Sciences. 91 (2021). https://doi.org/10.1007/s40011-021-01229-y.

[2] X. Luo, G. Cai, Y. Guo, C. Gao, W. Huang, Z. Zhang, H. Lu, K. Liu, J. Chen, X. Xiong, J. Lei, X. Zhou, J. Wang, Y. Liu, Exploring Marine-Derived Ascochlorins as Novel Human Dihydroorotate Dehydrogenase Inhibitors for Treatment of Triple-Negative Breast Cancer, Journal of Medicinal Chemistry. 64 (2021). https://doi.org/10.1021/acs.jmedchem.1c01402.

[3] E.B.G. Jones, S. Suetrong, J. Sakayaroj, A.H. Bahkali, M.A. Abdel-Wahab, T. Boekhout, K.L. Pang, Classification of marine Ascomycota, Basidiomycota, Blastocladiomycota and Chytridiomycota, Fungal Diversity. 73 (2015). https://doi.org/10.1007/s13225-015-0339-4.

[4] L. Panno, M. Bruno, S. Voyron, A. Anastasi, G. Gnavi, L. Miserere, G.C. Varese, Diversity, ecological role and potential biotechnological applications of marine fungi associated to the seagrass Posidonia oceanica, New Biotechnology. 30 (2013). https://doi.org/10.1016/j.nbt.2013.01.010.

[5] E.B.G. Jones, S. Suetrong, J. Sakayaroj, A.H. Bahkali, M.A. Abdel-Wahab, T. Boekhout, K.L. Pang, Classification of marine Ascomycota, Basidiomycota, Blastocladiomycota and Chytridiomycota, Fungal Diversity. 73 (2015). https://doi.org/10.1007/s13225-015-0339-4.

[6] J. Cao, X.M. Li, L.H. Meng, B. Konuklugil, X. Li, H.L. Li, B.G. Wang, Isolation and characterization of three pairs of indolediketopiperazine enantiomers containing infrequent N-methoxy substitution from the marine algal-derived endophytic fungus Acrostalagmus luteoalbus TK-43, Bioorganic Chemistry. 90 (2019). https://doi.org/10.1016/j.bioorg.2019.103030.

[7] S.T. Fang, F.P. Miao, X.H. Liu, Y.P. Song, N.Y. Ji, Two new tricycloalternarene acids from the marine-derived fungus Alternaria alternata ICD5-11, Phytochemistry Letters. 23 (2018). https://doi.org/10.1016/j.phytol.2017.12.009.

[8] J.M. Wong Chin, D. Puchooa, T. Bahorun, R. Jeewon, Antimicrobial properties of marine fungi from sponges and brown algae of Mauritius, Mycology. 12 (2021). https://doi.org/10.1080/21501203.2021.1895347.

[9] A. Poli, E. Bovio, L. Ranieri, G.C. Varese, V. Prigione, Fungal Diversity in the Neptune Forest: Comparison of the Mycobiota of Posidonia oceanica, Flabellia petiolata, and Padina pavonica, Frontiers in Microbiology. 11 (2020). https://doi.org/10.3389/fmicb.2020.00933.

[10] Marine Athrinium spp. Isolated from Sargassum sp. (Brown Algae) in Jeju Island and Unrecorded Species in Korea, The Korean Journal of Mycology. 44 (2016). https://doi.org/10.4489/kjm.2016.44.4.259.

[11] L. Garzoli, A. Poli, V. Prigione, G. Gnavi, G.C. Varese, Peacock's tail with a fungal cocktail: first assessment of the mycobiota associated with the brown alga Padina pavonica, Fungal Ecology. 35 (2018) 87–97. https://doi.org/10.1016/j.funeco.2018.05.005.

[12] L.M.Y. Mitchison-Field, J.M. Vargas-Muñiz, B.M. Stormo, E.J.D. Vogt, S. van Dierdonck, J.F. Pelletier, C. Ehrlich, D.J. Lew, C.M. Field, A.S. Gladfelter, Unconventional Cell Division Cycles from Marine-Derived Yeasts, Current Biology. 29 (2019). https://doi.org/10.1016/j.cub.2019.08.050.

[13] G. Gnavi, L. Garzoli, A. Poli, V. Prigione, G. Burgaud, G.C. Varese, The culturable mycobiota of Flabellia petiolata: First survey of marine fungi associated to a Mediterranean green alga, PLoS ONE. 12 (2017). https://doi.org/10.1371/journal.pone.0175941.

[14] E. Bovio, G. Gnavi, V. Prigione, F. Spina, R. Denaro, M. Yakimov, R. Calogero, F. Crisafi, G.C. Varese, The culturable mycobiota of a Mediterranean marine site after an oil spill: Isolation, identification and potential application in bioremediation, Science of the Total Environment. 576 (2017) 310–318. https://doi.org/10.1016/j.scitotenv.2016.10.064.

[15] J. Peng, X. Zhang, W. Wang, T. Zhu, Q. Gu, D. Li, Austalides S-U, new meroterpenoids from the sponge-derived fungus Aspergillus aureolatus HDN14-107, Marine Drugs. 14 (2016). https://doi.org/10.3390/md14070131.

[16] W. Sun, W. Wu, X. Liu, D.A. Zaleta-Pinet, B.R. Clark, Bioactive compounds isolated from marine-derived microbes in China: 2009-2018, Marine Drugs. 17 (2019). https://doi.org/10.3390/md17060339.

[17] X. Du, D. Liu, J. Huang, C. Zhang, P. Proksch, W. Lin, Polyketide derivatives from the sponge associated fungus Aspergillus europaeus with antioxidant and NO inhibitory activities, Fitoterapia. 130 (2018). https://doi.org/10.1016/j.fitote.2018.08.030.

[18] J. Xu, M. Yi, L. Ding, S. He, A review of anti-inflammatory compounds from marine fungi, 2000-2018, Marine Drugs. 17 (2019). https://doi.org/10.3390/md17110636.

[19] E. Bovio, L. Garzoli, A. Poli, V. Prigione, D. Firsova, G.P. McCormack, G.C. Varese, The culturable mycobiota associated with three Atlantic sponges, including two new species: Thelebolus balaustiformis and T. spongiae, Fungal Systematics and Evolution. 1 (2018). https://doi.org/10.3114/fuse.2018.01.07.

[20] E. Cohen, L. Koch, K.M. Thu, Y. Rahamim, Y. Aluma, M. Ilan, O. Yarden, S. Carmeli, Novel terpenoids of the fungus Aspergillus insuetus isolated from the Mediterranean sponge Psammocinia sp. collected along the coast of Israel, in: Bioorganic and Medicinal Chemistry, 2011. https://doi.org/10.1016/j.bmc.2011.05.045.

[21] E. Bovio, L. Garzoli, A. Poli, V. Prigione, D. Firsova, G.P. McCormack, G.C. Varese, The culturable mycobiota associated with three Atlantic sponges, including two new species: Thelebolus balaustiformis and T. spongiae, Fungal Systematics and Evolution. 1 (2018). https://doi.org/10.3114/fuse.2018.01.07.

[22] C. Raghukumar, S. Raghukumar, Barotolerance of fungi isolated from deep-sea sediments of the Indian Ocean, Aquatic Microbial Ecology. 15 (1998). https://doi.org/10.3354/ame015153.

[23] Y. Khambhaty, K. Mody, S. Basha, B. Jha, Kinetics, equilibrium and thermodynamic studies on biosorption of hexavalent chromium by dead fungal biomass of marine Aspergillus niger, Chemical Engineering Journal. 145 (2009). https://doi.org/10.1016/j.cej.2008.05.002.

[24] Y.M. Lee, M.J. Kim, H. Li, P. Zhang, B. Bao, K.J. Lee, J.H. Jung, Marine-Derived Aspergillus Species as a Source of Bioactive Secondary Metabolites, Marine Biotechnology. 15 (2013). https://doi.org/10.1007/s10126-013-9506-3.

[25] S. Dobretsov, H. Al-Shibli, S.S.N. Maharachchikumbura, A.M. Al-Sadi, The presence of marine filamentous fungi on a copper-based antifouling paint, Applied Sciences (Switzerland). 11 (2021). https://doi.org/10.3390/app11188277.

[26] S. Lee, M.S. Park, Y.W. Lim, Diversity of marine-derived Aspergillus from tidal mudflats and Sea Sand in Korea, Mycobiology. 44 (2016). https://doi.org/10.5941/MYCO.2016.44.4.237.

[27] P. Marchese, L. Garzoli, G. Gnavi, E. O'Connell, A. Bouraoui, M. Mehiri, J.M. Murphy, G.C. Varese, Diversity and bioactivity of fungi associated with the marine sea cucumber Holothuria poli: disclosing the strains potential for biomedical applications, Journal of Applied Microbiology. 129 (2020). https://doi.org/10.1111/jam.14659.

[28] F.Y. Du, X.M. Li, Z.C. Sun, L.H. Meng, B.G. Wang, Secondary Metabolites with Agricultural Antagonistic Potentials from Beauveria felina, a Marine-Derived Entomopathogenic Fungus, Journal of Agricultural and Food Chemistry. 68 (2020). https://doi.org/10.1021/acs.jafc.0c05696.

[29] J. Álvarez-Barragán, C. Cravo-Laureau, L.Y. Wick, R. Duran, Fungi in PAH-contaminated marine sediments: Cultivable diversity and tolerance capacity towards PAH, Marine Pollution Bulletin. 164 (2021). https://doi.org/10.1016/j.marpolbul.2021.112082.

[30] P. Marchese, L. Garzoli, R. Young, L. Allcock, F. Barry, M. Tuohy, M. Murphy, Fungi populate deep-sea coral gardens as well as marine sediments in the Irish Atlantic Ocean, Environmental Microbiology. 23 (2021) 4168–4184. https://doi.org/10.1111/1462-2920.15560.

[31] D. Greetham, A.S. Zaky, C. Du, Exploring the tolerance of marine yeast to inhibitory compounds for improving bioethanol production, Sustainable Energy and Fuels. 3 (2019). https://doi.org/10.1039/c9se00029a.

[32] L. Wang, Z. Chi, L. Yue, Z. Chi, D. Zhang, Occurrence and diversity of Candida genus in marine environments, Journal of Ocean University of China. 7 (2008). https://doi.org/10.1007/s11802-008-0416-3.

[33] X. Zou, Y. Wei, K. Dai, F. Xu, H. Wang, X. Shao, Yeasts from intertidal zone marine sediment demonstrate antagonistic activities against Botrytis cinerea in vitro and in strawberry fruit, Biological Control. 158 (2021). https://doi.org/10.1016/j.biocontrol.2021.104612.

[34] L. Panno, M. Bruno, S. Voyron, A. Anastasi, G. Gnavi, L. Miserere, G.C. Varese, Diversity, ecological role and potential biotechnological applications of marine fungi associated to the seagrass Posidonia oceanica, New Biotechnology. 30 (2013). https://doi.org/10.1016/j.nbt.2013.01.010.

[35] C.M. Cui, X.M. Li, C.S. Li, P. Proksch, B.G. Wang, Cytoglobosins A-G, cytochalasans from a marine-derived endophytic Fungus, chaetomium globosum QEN-14, Journal of Natural Products. 73 (2010). https://doi.org/10.1021/np900569t.

[36] G. le Goff, P. Lopes, G. Arcile, P. Vlachou, E. van Elslande, P. Retailleau, J.F. Gallard, M. Weis, Y. Benayahu, N. Fokialakis, J. Ouazzani, Impact of the Cultivation Technique on the Production of Secondary Metabolites by Chrysosporium lobatum TM-237-S5, Isolated from the Sponge Acanthella cavernosa, Marine Drugs. 17 (2019). https://doi.org/10.3390/md17120678.

[37] P. Marchese, L. Garzoli, R. Young, L. Allcock, F. Barry, M. Tuohy, M. Murphy, Fungi populate deep-sea coral gardens as well as marine sediments in the Irish Atlantic Ocean, Environmental Microbiology. 23 (2021) 4168–4184. https://doi.org/10.1111/1462-2920.15560.

[38] G. Cecchi, L. Cutroneo, S. di Piazza, M. Capello, M. Zotti, Culturable fungi from dredged and marine sediments from six ports studied in the framework of the SEDITERRA Project, Journal of Soils and Sediments. 21 (2021). https://doi.org/10.1007/s11368-021-02884-4.

[39] A. Maamar, M.E. Lucchesi, S. Debaets, N.N. van Long, M. Quemener, E. Coton, M. Bouderbala, G. Burgaud, A. Matallah-Boutiba, Highlighting the crude oil bioremediation potential of marine fungi isolated from the Port of Oran (Algeria), Diversity (Basel). 12 (2020). https://doi.org/10.3390/D12050196.

[40] Z. Fan, Z.H. Sun, H.X. Liu, Y.C. Chen, H.H. Li, W.M. Zhang, Perangustols A and B, a pair of new azaphilone epimers from a marine sediment-derived fungus Cladosporium perangustm FS62, Journal of Asian Natural Products Research. 18 (2016). https://doi.org/10.1080/10286020.2016.1181623.

[41] P. Zalar, G.S. de Hoog, H.J. Schroers, P.W. Crous, J.Z. Groenewald, N. Gunde-Cimerman, Phylogeny and ecology of the ubiquitous saprobe Cladosporium sphaerospermum, with descriptions of seven new species from hypersaline environments, Studies in Mycology. 58 (2007). https://doi.org/10.3114/sim.2007.58.06.

[42] A. Patyshakuliyeva, D.L. Falkoski, A. Wiebenga, K. Timmermans, R.P. de Vries, Macroalgae derived fungi have high abilities to degrade algal polymers, Microorganisms. 8 (2020). https://doi.org/10.3390/microorganisms8010052.

[43] G.H. Yu, G.W. Wu, T.J. Zhu, Q.Q. Gu, D.H. Li, Cladosins F and G, two new hybrid polyketides from the deep-sea-derived Cladosporium sphaerospermum 2005-01-E3, Journal of Asian Natural Products Research. 17 (2015). https://doi.org/10.1080/10286020.2014.940330.

[44] L. Garzoli, G. Gnavi, F. Tamma, S. Tosi, G.C. Varese, A.M. Picco, Sink or swim: Updated knowledge on marine fungi associated with wood substrates in the Mediterranean Sea and hints about their potential to remediate hydrocarbons, Progress in Oceanography. 137 (2015). https://doi.org/10.1016/j.pocean.2015.05.028.

[45] V.N. Gonçalves, G.A. Vitoreli, G.C.A. de Menezes, C.R.B. Mendes, E.R. Secchi, C.A. Rosa, L.H. Rosa, Taxonomy, phylogeny and ecology of cultivable fungi present in seawater gradients across the Northern Antarctica Peninsula, Extremophiles. 21 (2017). https://doi.org/10.1007/s00792-017-0959-6.

[46] B. Valderrama, J.J. Ruiz, M.S. Gutiérrez, K. Alveal, M. Caruffo, M. Oliva, H. Flores, A. Silva, M. Toro, A. Reyes-Jara, P. Navarrete, Cultivable yeast microbiota from the marine fish species genypterus chilensis and seriolella violacea, Journal of Fungi. 7 (2021). https://doi.org/10.3390/jof7070515.

[47] G. Greco, G. Cecchi, S. di Piazza, L. Cutroneo, M. Capello, M. Zotti, Fungal characterisation of a contaminated marine environment: the case of the Port of Genoa (North-Western Italy), Webbia. 73 (2018). https://doi.org/10.1080/00837792.2017.1417964.

[48] V. Nenkep, K. Yun, B.W. Son, Oxysporizoline, an antibacterial polycyclic quinazoline alkaloid from the marine-mudflat-derived fungus Fusarium oxysporum, Journal of Antibiotics. 69 (2016). https://doi.org/10.1038/ja.2015.137.

[49] Samuel, Prabakaran, Antibacterial Activity of Marine derived Fungi Collected from South East Coast of Tamilnadu, India, 2011. http://scholarsresearchlibrary.com/archive.html.

[50] R. Fotedar, A. Kolecka, T. Boekhout, J.W. Fell, A. Al-Malki, A. Zeyara, M. al Marri, Fungal diversity of the hypersaline Inland Sea in Qatar, Botanica Marina. 61 (2018) 595–609. https://doi.org/10.1515/bot-2018-0048.

[51] M.M. Francis, V. Webb, G.C. Zuccarello, Marine yeast biodiversity on seaweeds in New Zealand waters, New Zealand Journal of Botany. 54 (2016) 30–47. https://doi.org/10.1080/0028825X.2015.1103274.

[52] Minegishi H, Usami R, Abe F, Phylogenetic analysis of pectin degrading yeasts from deep-sea environments, 2006.

[53] G.M. Cabrera, M. Butler, M.A. Rodriguez, A. Godeas, R. Haddad, M.N. Eberlin, A sorbicillinoid urea from an intertidal Paecilomyces marquandii, Journal of Natural Products. 69 (2006). https://doi.org/10.1021/np060315d.

[54] A. Poli, E. Bovio, L. Ranieri, G.C. Varese, V. Prigione, Fungal Diversity in the Neptune Forest: Comparison of the Mycobiota of Posidonia oceanica, Flabellia petiolata, and Padina pavonica, Frontiers in Microbiology. 11 (2020). https://doi.org/10.3389/fmicb.2020.00933.

[55] L. Garzoli, A. Poli, V. Prigione, G. Gnavi, G.C. Varese, Peacock's tail with a fungal cocktail: first assessment of the mycobiota associated with the brown alga Padina pavonica, Fungal Ecology. 35 (2018) 87–97. https://doi.org/10.1016/j.funeco.2018.05.005.

[56] M.R.Z. Passarini, C. Santos, N. Lima, R.G.S. Berlinck, L.D. Sette, Filamentous fungi from the Atlantic marine sponge Dragmacidon reticulatum, Archives of Microbiology. 195 (2013). https://doi.org/10.1007/s00203-012-0854-6.

[57] G. Greco, G. Cecchi, S. di Piazza, L. Cutroneo, M. Capello, M. Zotti, Fungal characterisation of a contaminated marine environment: the case of the Port of Genoa (North-Western Italy), Webbia. 73 (2018). https://doi.org/10.1080/00837792.2017.1417964.

[58] X.W. Wang, F.Y. Bai, K. Bensch, M. Meijer, B.D. Sun, Y.F. Han, P.W. Crous, R.A. Samson, F.Y. Yang, J. Houbraken, Phylogenetic re-evaluation of Thielavia with the introduction of a new family Podosporaceae, Studies in Mycology. 93 (2019). https://doi.org/10.1016/j.simyco.2019.08.002.

[59] A. Poli, E. Bovio, L. Ranieri, G.C. Varese, V. Prigione, News from the sea: A new genus and seven new species in the pleosporalean families roussoellaceae and thyridariaceae, Diversity (Basel). 12 (2020). https://doi.org/10.3390/D12040144.

[60] W. Wang, S. Li, Z. Chen, Z. Li, Y. Liao, J. Chen, Secondary Metabolites Produced by the Deep-Sea-Derived Fungus Engyodontium album, Chemistry of Natural Compounds. 53 (2017). https://doi.org/10.1007/s10600-017-1957-8.

[61] L.A. Giddings, D.J. Newman, Extremophilic Fungi from Marine Environments: Underexplored Sources of Antitumor, Anti-Infective and Other Biologically Active Agents, Marine Drugs. 20 (2022). https://doi.org/10.3390/md20010062.

[62] L. Garzoli, A. Poli, V. Prigione, G. Gnavi, G.C. Varese, Peacock's tail with a fungal cocktail: first assessment of the mycobiota associated with the brown alga Padina pavonica, Fungal Ecology. 35 (2018) 87–97. https://doi.org/10.1016/j.funeco.2018.05.005.

[63] Q. Zhang, B. Yang, F. Li, M. Liu, S. Lin, J. Wang, Y. Xue, H. Zhu, W. Sun, Z. Hu, Y. Zhang, Mycophenolic acid derivatives with immunosuppressive activity from the coral-derived fungus penicillium bialowiezense, Marine Drugs. 16 (2018). https://doi.org/10.3390/md16070230.

[64] E. Bovio, E. Sfecci, A. Poli, G. Gnavi, V. Prigione, T. Lacour, M. Mehiri, G.C. Varese, The culturable mycobiota associated with the Mediterranean sponges Aplysina cavernicola, Crambe crambe and Phorbas tenacior, FEMS Microbiology Letters. 366 (2020). https://doi.org/10.1093/femsle/fnaa014.

[65] D. Reeb, P.B. Best, A. Botha, K.J. Cloete, M. Thornton, M. Mouton, Fungi associated with the skin of a southern right whale (Eubalaena australis) from South Africa, Mycology. 1 (2010). https://doi.org/10.1080/21501203.2010.492531.

[66] K. Takahashi, K. Sakai, Y. Nagano, S. Orui Sakaguchi, A.O. Lima, V.H. Pellizari, M. Iwatsuki, K. Takishita, K. Nonaka, K. Fujikura, S. Omura, Cladomarine, a new anti-saprolegniasis compound isolated from the deep-sea fungus, Penicillium coralligerum YK-247, Journal of Antibiotics. 70 (2017). https://doi.org/10.1038/ja.2017.58.

[67] J. Álvarez-Barragán, C. Cravo-Laureau, L.Y. Wick, R. Duran, Fungi in PAH-contaminated marine sediments: Cultivable diversity and tolerance capacity towards PAH, Marine Pollution Bulletin. 164 (2021). https://doi.org/10.1016/j.marpolbul.2021.112082.

[68] Y.H. Li, X.M. Li, X. Li, S.Q. Yang, X.S. Shi, H.L. Li, B.G. Wang, Antibacterial Alkaloids and Polyketide Derivatives from the Deep Sea-Derived Fungus Penicillium cyclopium SD-413, Mar Drugs. 18 (2020). https://doi.org/10.3390/md18110553.

[69] A.I. Khusnullina, E.N. Bilanenko, A. v. Kurakov, Microscopic Fungi of White Sea Sediments, Contemporary Problems of Ecology. 11 (2018). https://doi.org/10.1134/S1995425518050062.

[70] R. Nicoletti, A. Trincone, Bioactive compounds produced by strains of Penicillium and Talaromyces of marine origin, Marine Drugs. 14 (2016). https://doi.org/10.3390/md14020037.

[71] L.N. Zhou, T.J. Zhu, S.X. Cai, Q.Q. Gu, D.H. Li, Three new indole-containing diketopiperazine alkaloids from a deep-ocean sediment derived fungus Penicillium griseofulvum, Helvetica Chimica Acta. 93 (2010). https://doi.org/10.1002/hlca.200900443.

[72] S.Q. Yang, A. Mándi, X.M. Li, H. Liu, X. Li, S. Balázs Király, T. Kurtán, B.G. Wang, Separation and configurational assignment of stereoisomeric phenalenones from the marine mangrove-derived fungus Penicillium herquei MA-370, Bioorganic Chemistry. 106 (2021). https://doi.org/10.1016/j.bioorg.2020.104477.

[73] H. al Nasrawi, Biodegradation of Crude Oil by Fungi Isolated from Gulf of Mexico, Journal of Bioremediation & Biodegradation. 03 (2012). https://doi.org/10.4172/2155-6199.1000147.

[74] M. da Luz Calado, J. Silva, C. Alves, P. Susano, D. Santos, J. Alves, A. Martins, H. Gaspar, R. Pedrosa, M.J. Campos, Marine endophytic fungi associated with Halopteris scoparia (Linnaeus) Sauvageau as producers of bioactive secondary metabolites with potential dermocosmetic application, PLoS ONE. 16 (2021). https://doi.org/10.1371/journal.pone.0250954.

[75] T.P.T. Hoang, C. Roullier, G. Genta-Jouve, M.C. Boumard, T. Robiou du Pont, H. Nazih, Y.F. Pouchus, O. Grovel, C25 steroids from the marine mussel-derived fungus Penicillium ubiquetum MMS330, Phytochemistry Letters. 34 (2019). https://doi.org/10.1016/j.phytol.2019.09.002.

[76] Z. Paz, M. Komon-Zelazowska, I.S. Druzhinina, M.M. Aveskamp, A. Shnaiderman, Y. Aluma, S. Carmeli, M. Ilan, O. Yarden, Diversity and potential antifungal properties of fungi associated with a Mediterranean sponge, Fungal Diversity. 42 (2010). https://doi.org/10.1007/s13225-010-0020-x.

[77] G. Carr, W. Tay, H. Bottriell, S.K. Andersen, A.G. Mauk, R.J. Andersen, Plectosphaeroic acids A, B, and C, Lndoleamine 2,3-dioxygenase inhibitors produced in culture by a marine isolate of the fungus plectosphaerella cucumerina, Organic Letters. 11 (2009). https://doi.org/10.1021/ol900972j.

[78] A.K. Vala, Intra-and extracellular biosynthesis of gold nanoparticles by a marine-derived fungus Rhizopus oryzae, Synthesis and Reactivity in Inorganic, Metal-Organic and Nano-Metal Chemistry. 44 (2014). https://doi.org/10.1080/15533174.2013.799492.

[79] A.D. Pilevneli, S.S. Ebada, B. Kaşkatepe, B. Konuklugil, Penicacids H-J, three new mycophenolic acid derivatives from the marine-derived fungus: Rhizopus oryzae, RSC Advances. 11 (2021). https://doi.org/10.1039/d1ra07196c.

[80] C. Kaewkrajay, S. Putchakarn, S. Limtong, Cultivable yeasts associated with marine sponges in the Gulf of Thailand, South China Sea, Antonie van Leeuwenhoek, International Journal of General and Molecular Microbiology. 114 (2021). https://doi.org/10.1007/s10482-021-01518-6.

[81] A. Poli, A. Vizzini, V. Prigione, G.C. Varese, Basidiomycota isolated from the Mediterranean Sea – Phylogeny and putative ecological roles, Fungal Ecology. 36 (2018) 51–62. https://doi.org/10.1016/j.funeco.2018.09.002.

[82] C.F. Chang, C.F. Lee, K.Y. Lin, S.M. Liu, Diversity of yeasts associated with the sea surface microlayer and underlying water along the northern coast of Taiwan, Research in Microbiology. 167 (2016). https://doi.org/10.1016/j.resmic.2015.08.005.

[83] Y. Gao, X. Du, W. Xu, R. Fan, X. Zhang, S. Yang, X. Chen, J. Lv, Z. Luo, Fungal Diversity in Deep Sea Sediments from East Yap Trench and Their Denitrification Potential, Geomicrobiology Journal. (2020). https://doi.org/10.1080/01490451.2020.1789778.

[84] M. Gadanho, J.M. Almeida, J. Paulo, S. Sampaiõ, S. Sampaiõˆ´, Assessment of yeast diversity in a marine environment in the south of portugal by microsatellite-primed PCR, n.d. www.crem.fct.unl.pt.

[85] E. Breyer, M. Böhm, M. Reitbauer, C. Amano, M. Heitger, F. Baltar, Autofluorescence is a common trait in different oceanic fungi, Journal of Fungi. 7 (2021). https://doi.org/10.3390/jof7090709.

[86] M.M. Francis, V. Webb, G.C. Zuccarello, Marine yeast biodiversity on seaweeds in New Zealand waters, New Zealand Journal of Botany. 54 (2016) 30–47. https://doi.org/10.1080/0028825X.2015.1103274.

[87] J.W. Fell, Sakaguchia Y: Yamada, Maeda & Mikata (1994), in: The Yeasts, Elsevier, 2011: pp. 1541–1544. https://doi.org/10.1016/B978-0-444-52149-1.00128-2.

[88] W. Guo, S. Wang, N. Li, F. Li, T. Zhu, Q. Gu, P. Guo, D. Li, Saroclides A and B, Cyclic Depsipeptides from the Mangrove-Derived Fungus Sarocladium kiliense HDN11-112, Journal of Natural Products. 81 (2018). https://doi.org/10.1021/acs.jnatprod.7b00644.

[89] K.C. Hu, M.Y. Xu, H.J. Li, J. Yuan, G. Tang, J. Xu, D.P. Yang, W.J. Lan, Discovery of aromadendrane anologues from the marine-derived fungus: Scedosporium dehoogii F41-4 by NMR-guided isolation, RSC Advances. 6 (2016). https://doi.org/10.1039/c6ra21142a.

[90] I. Risnasari, R. Batubara, A. Nuryawan, F.A. Lubis, O. Firnadi, M.A. Maulana, Deterioration of wood exposed to mangrove ecosystem, in: IOP Conference Series: Earth and Environmental Science, 2021. https://doi.org/10.1088/1755-1315/782/3/032031.

[91] A.L. Grunwald, C. Cartmell, R.G. Kerr, Auyuittuqamides A-D, Cyclic Decapeptides from Sesquicillium microsporum RKAG 186 Isolated from Frobisher Bay Sediment, Journal of Natural Products. 84 (2021). https://doi.org/10.1021/acs.jnatprod.0c00966.

[92] M. Venkatachalam, L. Gérard, C. Milhau, F. Vinale, L. Dufossé, M. Fouillaud, Salinity and temperature influence growth and pigment production in the marine-derived fungal strain talaromyces albobiverticillius 30548, Microorganisms. 7 (2019). https://doi.org/10.3390/microorganisms7010010.

[93] J. xing Li, X. xin Lei, Y. hong Tan, Y. hong Liu, B. Yang, Y. qiu Li, Two new bioactive polyphenols from the soft coral-derived fungus Talaromyces sp. SCSIO 041201, Natural Product Research. 35 (2021). https://doi.org/10.1080/14786419.2020.1836632.

[94] K. Kim, Y.M. Heo, S. Jang, H. Lee, S.L. Kwon, M.S. Park, Y.W. Lim, J.J. Kim, Diversity of Trichoderma spp. In marine environments and their biological potential for sustainable industrial applications, Sustainability (Switzerland). 12 (2020). https://doi.org/10.3390/su12104327.

[95] Y. Zhao, D. Liu, P. Proksch, D. Zhou, W. Lin, Truncateols O-V, further isoprenylated cyclohexanols from the sponge-associated fungus Truncatella angustata with antiviral activities, Phytochemistry. 155 (2018) 61–68. https://doi.org/10.1016/j.phytochem.2018.07.017.

[96] D. Xu, M. Luo, F. Liu, D. Wang, X. Pang, T. Zhao, L. Xu, X. Wu, M. Xia, X. Yang, Cytochalasan and Tyrosine-Derived Alkaloids from the Marine Sediment-Derived Fungus Westerdykella dispersa and Their Bioactivities, Scientific Reports. 7 (2017). https://doi.org/10.1038/s41598-017-12327-1.
